# Supplementary material for: Patterns of Intron Gain and Loss in Fungi
Source: PLoS Biol. 2004 Nov 30;2(12):e422. doi: 10.1371/journal.pbio.0020422 (PMC532390; doi:10.1371/journal.pbio.0020422)
Supplement: Table S1 — Also available at http://genes.mit.edu/NielsenEtAl/. (4.3 MB ZIP). [file pbio.0020422.st001.zip › NielsenEtAl/html/788.html]

AN2081.1.NCU04789.1.MG00448.1.FG08604.1


```
 CLUSTAL W (1.82) Multiple Sequence Alignments - Introns Inserted


Sequence 1: NCU04789.1	614 aa
Sequence 2: MG00448.1	619 aa
Sequence 3: FG08604.1	617 aa
Sequence 4: AN2081.1	615 aa
Alignment Length: 767 aa
Number Identitical Residues: 154 aa
Alignment Score (without introns) 8755


MG00448.1 	-------~-------MAIR~SGIALTSTYAVQQCARLLK~TVDDKSIRTELRLLQQELNE
NCU04789.1	MDALLAK~LGSHAVNYAIR~SGIAITSTYAVNQCSRLLK~TVDDKGVYTELKKLQKLLDS
FG08604.1 	MDEFLAT~VGVQAMRYAIR~SGIALTSSYAIGQCSRLLK~SVEDKAVHAELKSLQNQLDN
AN2081.1  	MDAFLAR0LTQQAMNYAIR2SGIAITASYAIRQSSRLLQ0--------------------
          	 .   :      :   *** ****:*::**: *.:***:                     

MG00448.1 	RIK0IISPAIDLIEFK2AGRGNVFLENAVPLAKGLRRSIVALGKRLEDVACAEEEA-QQH
NCU04789.1	KIK0IVSPAIDLIEFK2SGRGNVFLEAAVPLAKSLHRDIISLGKRLNDAATAEESS-RSD
FG08604.1 	KIK0IISPAIDLIEFK2SGRGNVFLESAAPLAKSLHKDIVSLGRRIEAVTTVNSSSNRVQ
AN2081.1  	---~VISPAIDMIELI2AARGNTSLESAVCLTKSLRLDIQSLGQRLATAAFSEEQSQKGG
          	    ::*****:**:  :.***. ** *. *:*.*: .* :**:*:  .:  :..:.:  

MG00448.1 	AREARARPRTPESQAAELRSIICEMKTLLGRIERDAHMLQFAISASGENLSTSLPPGVSP
NCU04789.1	GKKARMS----EAHHIELLTIIKEMKELLARIDREIPLIQLAITASGERMNTTVNAGISP
FG08604.1 	TKPLRVS-------RDDLLAIASGIKALLERIDREIPLMQLAISASGESLSTSLPANISP
AN2081.1  	KSPTDMS-----RSREMIKLIIQDIKRLLVRIEDAVPLMNLAITTSGAKLSTNLPATISP
          	                 :  *   :* ** **:    ::::**::**  :.*.: . :**

MG00448.1 	SRLMQASTFLSIGDTQFASDPYGPVQIGPSFTLSLYLLFMGHSKTETRARPAQGNALTTI
NCU04789.1	SRLMQASAFLSFGDSHFVNNPNRAVPIGPSFTLSLYMLFRGHSRLKSAS-----SVPATP
FG08604.1 	SRLLQASTLLTVGDTQFAQDPTRPVQIGPAFTLSLYMLFLGHASTLMPHASS--GVTDTT
AN2081.1  	SRLLQASTFLTAGDTQYSISPSQAVQIGPSFTLSMYMLFASHLRPHDEE-----------
          	***:***::*: **:::  .*  .* ***:****:*:** .*                  

MG00448.1 	NSGNPVNNSTDQVDSPYGFQEGERKPLWQEVVHKARVRLCRVPPDWKFDRERGYIPLPLP
NCU04789.1	STGG----KTWTREPSYGVGEGERKPIWQEVVHKARVRLVRTPLGWKFDKTRGYCPGESS
FG08604.1 	VSTNNHSSDSSSMGTSYGLGEGERKPLWQEVLHKTRVRICRTRRDTYFDSQRGYCTVPLE
AN2081.1  	---------------------GIRETTWKEVMHKARLKLRRVPMDMAVAP-GSQRPLQIP
          	                     * *:. *:**:**:*::: *.  .  .    .  .    

MG00448.1 	QSPN--RDQRAHSSYSYTAK-NEFSYHLEIIEDLDDGRLH---DGEGSF--FDDIPMAGI
NCU04789.1	DGPVNAYSAAVDSALSMLGRPDEYAYHLEIIEDLDDGRVH---DDD--------------
FG08604.1 	DYALPLPEATGG---------GEYAYHLEIIEDLDDGRVH---DDENDSQSYGDIPHAGI
AN2081.1  	ADAR----------------IDEFAYQILVVEDLHDGRVHAFDENESQPQSFEDIDAAGI
          	  .                  .*::*:: ::***.***:*: .:.:.. .:  .   :. 

MG00448.1 	RESIPVHHLSKIFYTNTG~RILNIGQELAPDNSPALLLKRDSTAPTPKQVIDDILQSMEE
NCU04789.1	---------------AAG1RLLNIGNAEEGDNNPVLLLKRDLHAPNPVQLKRQWYADPEQ
FG08604.1 	RESIPVHQLSKIFYTDTG~RILNIGSATDSEQRPVLLLKRDVQVGPPRSEHDDINGFIEE
AN2081.1  	REIIPIHQISKIFYADTG~RILNISTEGETNN-PVLLLKRDINAVPPRRMVERDMAGFGA
          	 .  .    :.   : :* *:***.     :: *.******  .  *             

MG00448.1 	AVTGADESDFQNEALMDS-QAELDLQILRESSESPSEEAPSPHDQRRPASCPVPKSSHSF
NCU04789.1	AEASEGETAYDTAAEDDDEQADIDAQLLAETKS--TELATAAEEGR-------LRKALGL
FG08604.1 	LDREGNDAALDSTSKNGD-QADIDKQLLGEFVRPAAEGPNISIPPSK------DYFKWKF
AN2081.1  	PHESEPED------EIDEIQAQLDAQLIGTTVN---TLNPSFHESS-------IPDQWRL
          	      :         .. **::* *::                               :

MG00448.1 	PKHLDPEWIALEVFAEIEDESNYSTEDGSSDDDPD---SSMSTAKSRSDVKGKRSSLDTK
NCU04789.1	PSHLDPEWLALEVFVESDDES-----DDDDDGDDK---SSIQSCKS---ATPRDKVVDSK
FG08604.1 	PVHLDPEWMALEVYEEQDDEEDPDEDDDDDSLDSARLDHPRMVSKSSTPHRGRRS-LDSR
AN2081.1  	PKDLDPEWIAFEVYNE-NDES-----DTESDDE-----------------TTKTPDPTEK
          	* .*****:*:**: * :**.     * ... :                   :      :

MG00448.1 	LMQQIRDISLR-----------------SGTTPPPHSGRE--------------SRKTVS
NCU04789.1	LIDQIRNVSLQPYPNTPPNQRKYVPSPLSSTSSLPTGGANGKQLFQTPAQVQGAQKEPNS
FG08604.1 	VVDQIKRLSLSS----------------PEKMSSLQAAMS----------------SPTE
AN2081.1  	MAQLSINADRE-----------------SRRTASPQPGKS------------------QS
          	: :     .                   .   .    . .                   .

MG00448.1 	PESFATRSPFGSITSSLSLLEMIIRLASLQEFQQTSHLSIPDHILTFFLEETATTGLSGE
NCU04789.1	MDSFVARSPFGAITSSLSLMEMLIRLCSLQEFQQTSHLSIPDHILTFFLEETSTTGLRGE
FG08604.1 	TEDFVARSPFGAITTSLSLMEMLIRLASLQEFQQTSHLSIPDHILTFFLEETSTTGLVGE
AN2081.1  	QPTTISNPLFNNIRTSLSLLETLLRLMSLQQFQQQSHLSISDELLNFFLEESSTTGAGGD
          	     :.. *. * :****:* ::** ***:*** *****.*.:*.*****::***  *:

MG00448.1 	D--RWKARSEAKKKVGFDPYHDAES-----------------------------------
NCU04789.1	E--KWDIRNEAKRRMGFDPYTDTPTKST--------------------------------
FG08604.1 	T--QWRARSEAKRRMGFDPYTDATTR----------------------------------
AN2081.1  	EQYRQRLRADARRRVGWDPYDESPVKRRGEDYQYSGTPEGMSIGYTRESSEIPYTPSERT
          	 . :   * :*::::*:*** ::     ... . :.:... : . : .::. . :.:. :

MG00448.1 	-----------------------------------------------------
NCU04789.1	-----------------------------------------------------
FG08604.1 	-----------------------------------------------------
AN2081.1  	RGYQLRSRENTPETPSQMRRSVSRAADLRRDSGTRGSSAHTHERQRRGSPLAH
          	 . .  : ..:..:.:.   : : ::.   .:.: .::: : . .  .:. :
```
